# A Rare Coincidence of Measles with Typhoid Fever

**DOI:** 10.7759/cureus.1630

**Published:** 2017-08-30

**Authors:** Omair ul haq Lodhi, Syed F Imam, Mahad Umer, Rizwan Zafar

**Affiliations:** 1 Shifa College of Medicine, Shifa International Hospital, Islamabad, Pakistan; 2 Department of Internal Medicine, Shifa International Hospital, Islamabad, Pakistan

**Keywords:** typhoid, measles, mmr, vaccination, coincidence, rare. enteric, fever

## Abstract

Typhoid, otherwise known as enteric fever, and measles both have a high incidence worldwide. However, a coincidence of both has been only documented twice previously in 1866 and 1949. We present a case of a 24-year-old male who presented with high-grade fever and diffuse abdominal tenderness. He was diagnosed with typhoid initially, but during the course of his illness, he developed a maculopapular rash and pathognomonic Koplik’s spots. Further investigations confirmed measles that was concurrent with typhoid. This highlights the importance of further comprehensive investigations even in diagnosed cases, and that overlapping symptoms should raise the clinical suspicion of concurrent diseases. One should always keep an open mind when assessing a patient, not just at the time of making a diagnosis but throughout the course of illness.

## Introduction

Typhoid, also known as enteric fever, is a systemic illness most commonly caused by Salmonella enterica serovar Typhi (S. typhi), a gram-negative bacterium. It is transmitted in humans by feco-oral route, and has an incubation period ranging from 7 to 14 days [1]. Typhoid has been reported to have a worldwide incidence ranging from 11.9 million to 26.9 million cases per year. The worldwide case fatality rate is 1% and the mortality ranges from 129,000 deaths to 161,000 per year [2]. The incidence in South East Asia was reported to be 575,407 cases in the year 2000 [3].

Measles, an airborne virus, caused by paramyxovirus group, is a highly transmissible disease [4]. The incidence of measles was reported to be 40 cases per million population in 2014 worldwide, and has a reported mortality rate of 114,900 deaths annually [5].

The last two cases of reported measles and typhoid coincidence in literature were in 1866 and 1949 [6-7]. We report an uncommon occasion in which our patient developed a rare conjunction of typhoid and measles.

## Case presentation

A 24-year-old male with no known comorbid was presented to the outpatient department with high-grade fever and diffuse abdominal pain since the past five days. The fever on presentation was 101 F but increased to 102.5-103.5 F which was persistent, not relieved by antipyretics, and associated with lethargy, chills, shivering and sweating. The abdominal pain was diffuse, 6/10 in intensity, relieved by defecation and associated with nausea, anorexia and bloating. There was no vomiting or changes in bowel movement.

On examination, the patient appeared ill, dehydrated, and pale. His pulse was 95 beats per minute, SpO2 was 97%, blood pressure (BP) was 110/60 mmHg, temperature was 103 F, and respiratory rate was 24 breaths per minute. Abdominal examination revealed diffuse abdominal tenderness with increased bowel sounds.

On admission, his initial workup was done and he received intravenous fluids and empirical antibiotics. Laboratory investigations are shown below in Table 1.

**Table 1:** Serial laboratory investigations. MCH: Mean corpuscular hemoglobin; MCHC: Mean corpuscular hemoglobin concentration; MCV: Mean corpuscular volume; RDW-CT: Red blood cell distribution width.

Laboratory Investigations:	Day 1	Day 2	Day 3	Day 4	Day 5	Day 6	Normal Values
White Blood Cell Count	5460	3570	2020	3280	4820	6350	(5500-11000/ul)
Red Blood Cell Count	5.14	5.14	5.01	4.70	5.02	5.13	(4.7-6.1 x 10^6^ cells/ul)
Hemoglobin	14.4	13.9	13.7	13.4	13.8	14.2	(13.5-17.5 g/dl)
Hematocrit	43	42.3	41	39	41	41	(38.8-50 % )
MCV	84	82.2	82	83	82	81	(80-96 fl)
MCH	28	27	27	29	28	28	(27-33 pg/cell)
MCHC	34	32.8	34	34	34	34	(33-36 g/dl)
RDW-CV	13	13	13	14	13	13	(11.5-14.5 % )
Platelets	217,000	229,000	195,000	167,000	148,000	213,000	(150,000-400,000/ul)
Neutrophils	81	79.7	76.3	74	61	41	(45-75 % )
Lymphocytes	8	10	13	14	27	43	(20-40 % )
Monocytes	10	12	8	6	10	12	(2-10 % )
Eosinophils	1	2	2	5	2	4	(0-6 % )
Basophils	0	0	0	0	0	0	(0-2 % )
Bands	0	0	1	1	0	0	(3-5 % )
Total Bilirubin	0.2	0.2	0.1	0.1	-	-	(0.3-1.9 mg/dl)
Alanine aminotransferase	273	213	207	186	-	-	(7-56 u/l)
Aspartate aminotransferase	206	217	223	257	-	-	(10-40 u/l)
Alkaline Phosphatase	126	144	128	118	-	-	(44-147 u/l)
Gamma GT	134	167	161	152	-	-	(9-48 u/l)

The patient was diagnosed with typhoid (enteric fever) and was started on intravenous ceftriaxone and continued intravenous fluids.

**Figure 1:**
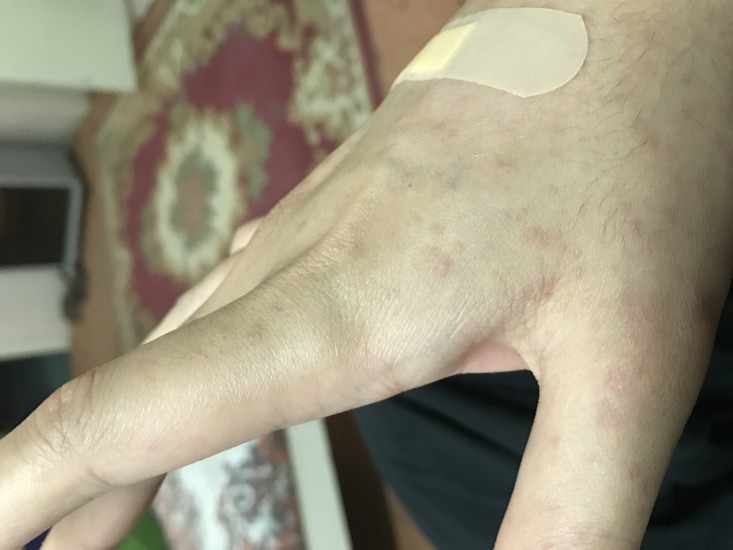
Erythematous maculopapular rash.

**Figure 2:**
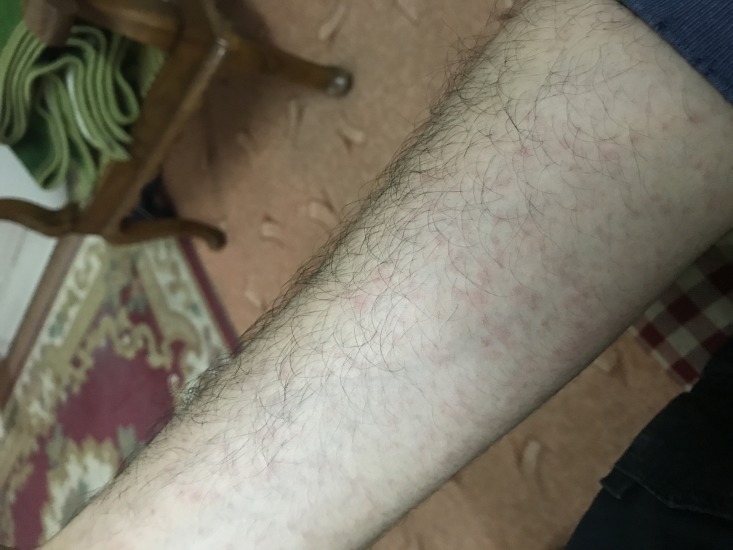
Erythematous maculopapular rash.

On day 2 of admission, the patient developed severe diarrhea, difficulty swallowing, pruritus and exanthematous maculopapular rash insidiously as shown in Figure 1 and Figure 2. The morbilliform rash started on the face and ultimately spread centrifugally towards the limbs from the trunk. In addition to the rash, the patient developed Koplik’s spots and conjunctivitis.

Further laboratory investigations are shown in Table 2.

**Table 2:** Serology report.

Serology	Malarial Parasite	Typhoid IgM	Typhoid IgG	Blood Culture	Dengue NS-1 antigen	Measles IgM	Measles IgG	CHIKV (RT-PCR)
Result	Negative	Positive	Negative	Salmonella Typhi	Negative	Positive	Negative	Negative

The patient was eventually diagnosed with measles superimposed over typhoid. Besides continuing previous management, he also received intravenous metronidazole, oral antipyretics, antihistamine and vitamin A supplements. After seven days of intensive therapy, the patient’s symptoms were relieved, and he was discharged in a stable condition with a follow-up plan of care.

## Discussion

Despite their high incidence rate separately, typhoid and measles have not been known to occur together, except for in two reported cases dating back to 1866 and 1949 [6-7]. Considering this rare occurrence, we document the clinical course of a 24-year-old male, who presented in a tertiary care setting with complaints of high-grade fever and diffuse abdominal pain since five days. The next day he started developing an erythematous maculopapular rash which then spread centrifugally and was also found to have Koplik’s spots.

Typhoid patients are typically febrile with diffuse abdominal pain and altered bowel habits. Nonspecific symptoms such as frontal headache, anorexia, lethargy, and dizziness may also be present, which is similar to our case. Examination may reveal relative bradycardia, rose spots, and tender hepatosplenomegaly. Whereas our patient only had relative bradycardia. Complications have been seen to increase after two weeks, which include gastrointestinal bleeding and intestinal perforation, 10% and 1-3% respectively in hospitalized patients, particularly if left untreated. Other complications may include cholecystitis, hepatitis, pneumonia, myocarditis, acute kidney injury, meningitis, and encephalopathy. Our patient, however, did not develop any complications [1].

Measles classically results in a febrile illness with concurrent morbilliform rash. The diagnostic criteria include three of the following:

1) Signs and symptoms consisting of fever, maculopapular rash on trunk and limbs, and one or more of the following: cough, Koplik’s spots, coryza or conjunctivitis;

2) Endemic area; and

3) MMR (Measles, Mumps and Rubella) vaccination status meaning incomplete vaccination series or unvaccinated patient.

Our patient, who was vaccinated in his childhood with the MMR vaccine, currently residing in an endemic area started developing all of the signs and symptoms of the measles criteria mentioned above, and continued to have manifestations of the preceding diagnosis, that is typhoid [8].

Complications of measles that are not uncommon include diarrhea (8%), pneumonia (6%) and otitis media (7%). In rare instances, patients may develop primary measles encephalitis which is the most common cause of mortality (0.1%), acute post infectious measles encephalomyelitis, measles inclusion body encephalitis, and subacute sclerosing panencephalitis. Our case developed none of these complications [9].

Typhoid usually presents with normal white blood cell count, eosinopenia, normochromic anemia, increased erythrocyte sedimentation rate, mild thrombocytopenia, increased alanine transferase and aspartate transferase. In our initial workup, the patient had neutrophilia (81%) and lymphopenia (8%). Gradually in the following days, a decreasing trend in neutrophils and a reciprocal increasing trend in lymphocytes were observed. The rise in lymphocytes corresponded with the onset of maculopapular rash. Additionally, an elevation in ALT (alanine aminotransferase) and AST (aspartate aminotransferase) were observed as shown in Table 1.

Typhoid can be diagnosed using different modalities. The gold standard is blood culture which is positive in 80% or above. Serological assays include antibody detection by Widal test, which can correctly diagnose 74% of blood culture positive patients, but has 14% false positive and 10% false negative results. Typhidot, a rapid serological test, detects IgM and IgG typhoid specific antibodies having a sensitivity between 67% and 98%, and a specificity between 73% and 100%. Less commonly used modalities include bone marrow aspirate culture (sensitivity greater than 80%), PCR (polymerase chain reaction) and ELISA (enzyme linked immunosorbent assay). On initial testing, typhidot was positive in our patient. Subsequently, his blood culture was positive for Salmonella typhi, hence confirming the diagnosis.

Our patient received intravenous ceftriaxone due to disease progression and severity. In the literature, recommended first line drugs for uncomplicated typhoid are trimethoprim-sulfamethoxazole, ampicillin, and chloramphenicol. Alternative drugs are extended spectrum cephalosporins, azithromycin, and fluoroquinolones that can be used individually or in combination depending on the severity of the disease and drug resistance [1]. Typhoid vaccine is available in both oral and injectable forms. However, it is not used commonly due to its short period of protection and complex administration regimens [2].

Classically, measles symptoms may resemble dengue virus, chikungunya, rubella virus, parvovirus B19, epstein bar virus, Streptococcus pyogenes or coxsackie virus so it is imperative to perform diagnostic laboratory tests. Serological assays were performed for disease with similar clinical presentation. The standard diagnostic test for measles infection is anti-measles virus IgM antibodies by ELISA in serum. Other than IgM serology, the most frequently used test for confirmation of acute virus is reverse transcriptase PCR. In our case, the anti-measles virus IgM antibodies were positive hence confirming measles [10].

The treatment of measles is primarily supportive. The mainstay is prevention using MMR vaccine. However, primary or secondary vaccine failure can result in future infections. Similarly, our case describes a patient who experienced vaccine failure, as his MMR vaccination was documented. He received supportive treatment with intravenous fluids for his dehydration, antihistamines for pruritus, and antipyretics for his fever [4].

## Conclusions

Typhoid and measles despite being vaccine preventable diseases have a high incidence worldwide. Even after vaccination during childhood, patients may present with primary or secondary vaccine failure. Therefore, if a patient presents with signs and symptoms of a previously vaccinated disease, further investigation is warranted. Also, it needs to be highlighted that the presentation of different diseases can overlap, thus one should always keep an open mind when assessing a patient, not just at the time of making a diagnosis, but throughout the course of the disease.
